# Neural Substrates for Semantic Memory of Familiar Songs: Is There an Interface between Lyrics and Melodies?

**DOI:** 10.1371/journal.pone.0046354

**Published:** 2012-09-28

**Authors:** Yoko Saito, Kenji Ishii, Naoko Sakuma, Keiichi Kawasaki, Keiichi Oda, Hidehiro Mizusawa

**Affiliations:** 1 Positron Medical Center, Tokyo Metropolitan Institute of Gerontology, Itabashi-ku, Tokyo, Japan; 2 Research Team for Promoting Independence of the Elderly, Tokyo Metropolitan Institute of Gerontology, Itabashi-ku, Tokyo, Japan; 3 Department of Neurology Neurological Science, Tokyo Medical Dental University, Graduate School of Medical Dental Science, Bunkyo-ku, Tokyo, Japan; Chiba University Center for Forensic Mental Health, Japan

## Abstract

Findings on song perception and song production have increasingly suggested that common but partially distinct neural networks exist for processing lyrics and melody. However, the neural substrates of song recognition remain to be investigated. The purpose of this study was to examine the neural substrates involved in the accessing “song lexicon” as corresponding to a representational system that might provide links between the musical and phonological lexicons using positron emission tomography (PET). We exposed participants to auditory stimuli consisting of familiar and unfamiliar songs presented in three ways: sung lyrics (song), sung lyrics on a single pitch (lyrics), and the sung syllable ‘la’ on original pitches (melody). The auditory stimuli were designed to have equivalent familiarity to participants, and they were recorded at exactly the same tempo. Eleven right-handed nonmusicians participated in four conditions: three familiarity decision tasks using song, lyrics, and melody and a sound type decision task (control) that was designed to engage perceptual and prelexical processing but not lexical processing. The contrasts (familiarity decision tasks versus control) showed no common areas of activation between lyrics and melody. This result indicates that essentially separate neural networks exist in semantic memory for the verbal and melodic processing of familiar songs. Verbal lexical processing recruited the left fusiform gyrus and the left inferior occipital gyrus, whereas melodic lexical processing engaged the right middle temporal sulcus and the bilateral temporo-occipital cortices. Moreover, we found that song specifically activated the left posterior inferior temporal cortex, which may serve as an interface between verbal and musical representations in order to facilitate song recognition.

## Introduction

Singing is one of the oldest cultural activities of human beings, one that combines a verbal component (lyrics) with a musical component (melody). Studies on the neural basis of song have thus focused on how these two components are processed in our brains. Evidence from behavioral studies [1] and from electrophysiological studies [2] has suggested that lyrics and melody are processed separately during detecting verbal and melodic errors in song. Lesion studies have also indicated dissociation between these components, as demonstrated by better performance on production of melody than on production of lyrics in nonfluent aphasic patients [3], [4] and by preservation of the recognizability of lyrics but not of melody in a music-agnosic patient [5]. In contrast, several behavioral and neurophysiological studies have reported that neural pathways involved in each domain interact at some stage of processing [6]–[10]. Over the last decade, evidence regarding song perception (listening or discrimination tasks) and song production (singing) has been accumulated using PET, fMRI and other modalities. The evidence supports the existence of an interactive relationship between the processing of lyrics and melody based on the common but partially distinct neural substrates of verbal and melodic processing. This interactivity has been observed in both song perception [11]–[14] and song production [11], [15]–[17].

For instance, Schön et al. [14] have investigated the neural networks involved in the song perception by fMRI. Participants listened to pairs of spoken words, vocalise (sung melody on the syllable ‘vi’), and sung words while performing a same-different task. They have shown a similar network including superior and middle temporal cortex and inferior frontal cortex for perception of speech, vocalise and song. Brown et al. [15] have examined neural correlation of sentence and melody generation using PET. In that study, participants listened to incomplete novel sentences or melodies that they were asked to complete by generating appropriate endings. They suggested that there were three stages of interaction between speech and music in the brain: sharing (primary auditory cortex and primary motor cortex), parallelism (superior temporal cortex and inferior frontal cortex for phonological generativity), or distinction (extrasylvian temporal lobe for semantic/syntax interface). It remains unclear, however, the neural mechanism implicated in the stage of recognition of familiar songs. In addition to considering previous findings, an examination of the neural relationships between verbal and melodic processes in song recognition requires rigorous consideration of each theoretical process stage mentioned below.

Based on lesion studies of musically impaired patients with selective brain damage, Peretz and Coltheart [18] proposed a modular functional model of music processing. This model postulates separate but interactive modules for the musical lexicon and the phonological lexicon. They defined the musical lexicon as a representational system that contains all representations of the specific musical phases to which one has been exposed during one's lifetime [18]–[20]. We use this model as a basis for understanding the processing of song that contains both musical and phonological information, which are usually tightly bound. In the present study, we use the term “song lexicon” as a conceptual representational system in which the musical lexicon and the phonological lexicon integrate and interact. Recognition of a sung familiar song consists of a set of computations that transform the acoustic waveform of the song into a representation that accesses the song lexicon [21]–[23]. In other words, recognition of a familiar song appears to include basic acoustic analysis, perceptual and phonological processing, access to the song lexicon (lexical access), selection of potential candidate matches in semantic memory, and integration [18], [19], [21], [24]–[26]. In this paper, we focus on the recognition of familiar songs, especially the processing stage of lexical lookup of songs, which takes place after the perceptual and phonological stages.

For verbal materials, numerous neuroimaging studies have reported that tasks involving semantic memory processes mainly activate the left hemisphere, focusing on areas such as the inferior frontal gyrus, the middle and inferior temporal gyri, the fusiform gyrus, the parahippocampal gyrus, the posterior cingulate gyrus, and the angular gyrus (for review, see [25]). Hickok and Poeppel have proposed a dual-stream model of speech processing, which nominates a dorsal stream that maps sound to articulation and a ventral stream that maps sound to meaning [21]. Recognition of familiar songs, including verbal components thereof, is predicted to recruit the ventral stream, which projects ventrolaterally toward the inferior posterior temporal cortex and serves as an interface between sound-based representations and lexical conceptual representations of speech. On the other hand, few studies on semantic memory of musical materials have been reported. Some studies have reported that regions activated during familiarity decision tasks involving melodies were located in the bilateral medial and orbital frontal regions, the left angular gyrus, the left anterior part of the temporal lobe [27], the bilateral anterior part of the temporal lobe, and the parahippocampal gyrus [28], and left temporal sulcus and left middle frontal gyrus [29]. For recognition of familiar tunes, Peretz et al. [22] have suggested that the right superior temporal sulcus contained musical lexical networks by comparing passive listening to familiar music relative to unfamiliar music. Investigating the semantic congruence of verbal materials (proverbs) and musical materials (classical melodies), Groussard et al. [30] reported that the two types of materials engage distinct neural networks compared with perceptual reference tasks. Furthermore, Groussard et al. [31] extended their study by introducing a familiarity judgment task for the two types of materials. They confirmed the distinction between the two types of materials in semantic memory based on results indicating that familiarity judgment of proverbs activated the left middle and inferior temporal gyri, whereas that of melodies mainly activated the bilateral superior temporal gyrus. However, it remains unclear how the verbal and melodic components of familiar songs are processed in the semantic memory system. To address this issue, we investigate the neural substrates of song recognition via direct comparisons between three types of sound stimuli: lyrics, melody, and song.

In order to compare these three sound types directly, it is necessary to control for attributes such as familiarity and emotional factors [24], [32], [33]. To this end, we investigated ratings of familiarity, age of acquisition, retrievability of lyrics and melody, and emotional factors associated with 100 Japanese children's songs in a preparatory study [34]. On the basis of these results, songs for use as auditory stimuli were chosen for the present study and synthesized in order to be as similar as possible in terms of familiarity, acoustical features (such as intensity, voice timbre, prosody, and duration), and temporal structures.

In addition to manipulating the auditory stimuli, we paid special attention to the design of the target task and the control task. First, in order to examine the neural substrates dedicated to lexical lookup of songs while minimizing the retrieval of associative memories, emotion, etc., we employed a familiarity decision task (decision between known and unknown) demanding that participants respond as quickly as possible. Second, in order to elucidate the neural networks involved in song lexical lookup beyond perceptual processing, a control task was designed that engages perceptual processing, monitoring of variations in phoneme and the pitch of auditory inputs, decisional processing, and motor processing, but not lexical access processing [18], [20], [21]. Based on previous neuroimaging research [15], [30], [31], we expected to see distinct neural pathways involved in lyrics and melody processing in the stage of the lexical lookup of songs. Our previous behavioral study demonstrated that the recognition of song is the fastest when song was presented in its entirety compared to when lyrics or melody was presented in isolation, even though the familiarity and other attributes of the stimuli were controlled [34]. Based on this finding, we hypothesized that an interface area may link verbal and melodic representation to facilitate song recognition.

## Methods

### Participants

Eleven native speakers of Japanese (11 men, mean age 20.8 years, range 20–23 years) participated in this study. All participants were right-handed, as confirmed by a modified version of the Edinburgh Handedness Inventory [35], and were free of any neurological or hearing impairments. The participants fulfilled the following two criteria: they had no professional musical education or training (mean 3.1 years of music lessons other than music education classes at primary and secondary school), and they were “common listeners” (i.e., not “music lovers,” who tend to listen to one specific type of music only; their time spent listening to music per day was 1.1 (SD = 0.74) hours.

### Ethics statement

This study is approved by the ethical committee of the Tokyo Metropolitan Institute of Gerontology. All participants gave written informed consent to participate in this study.

### Auditory stimuli

Twenty-four highly familiar songs and twenty-four unfamiliar songs were selected to serve as stimuli based on the results of our preparatory study of familiarity ratings of Japanese children's and traditional songs [34]. The mean familiarity rating on our five-point scale (1 = unfamiliar, 5 = highly familiar) was 4.69 for the familiar songs and 1.19 for the unfamiliar songs. The beginnings of the familiar songs were rated as equally familiar even when the lyrics or the melody was presented in isolation. Additionally, 30 songs with intermediate familiarity were used during a training session. A total of 234 sound stimuli were prepared.

**Figure 1:**
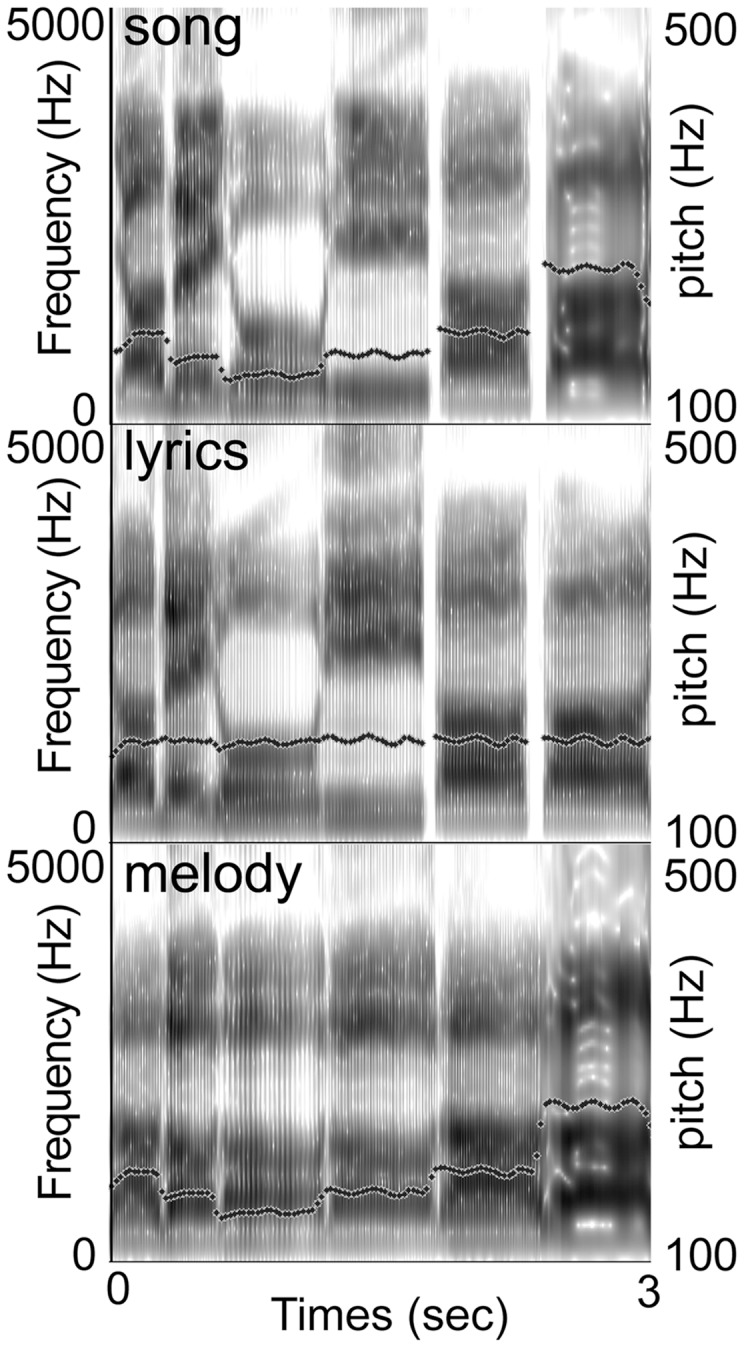
Acoustic analyses of three types of auditory stimuli: song, lyrics, and melody. Three images illustrate wide-band spectrograms of frequency information. Dotted lines represent pitch contours.

In the first type of sound stimuli (song), original lyrics were sung to the original melody. In the second type of sound stimuli (lyrics), the original lyrics were sung using the original rhythm, but on a single pitch (G3, 196 Hz). In the third type of sound stimuli (melody), the syllable “la” was sung to the original melody and using the original rhythm. All stimuli were generated by the VOCALOID voice-synthesizing software (YAMAHA, Inc., Tokyo, Japan) in order to make the three types of sound stimuli as similar as possible in terms of acoustical features, such as intensity, voice timbre, prosody, and duration. As a result, the three types of sound stimuli had exactly the same temporal features, such as tempo, rhythm, and duration of notes (Figure 1). None of the stimuli contained instrumental or choral accompaniment. The auditory stimuli were digital music files with 16-bit depth, 44,100-Hz sampling rate, and mean loudness of 75.1 dB SPL. The warning stimulus was a pure tone (sine wave, 500-Hz frequency, 500-ms duration). All sound stimuli were presented using E-Prime (Psychology Software Tools, Inc., Pittsburgh, USA).

We checked the clearness of and participants' feelings of discomfort with the synthetic voices compared to natural human voices in the pre-experimental study, which tested 12 participants (12 men, mean age = 21, SD = 2). The results showed that the clearness of and participants' feelings of discomfort with the synthetic voices were not significantly different from values obtained using human voice sounds (*t* = 1.02, *p*>0.13, paired *t*-test).

### Procedure

Prior to each PET scanning session, the participant completed a short practice session in which five sound stimuli were presented. During PET scanning, there were two decision tasks: a familiarity decision (FamD) and a sound-type decision (which was a perceptive control task [Control]). There were three sound type conditions in which FamD task were made labeled as Song, Lyrics, and Melody. For each sound type condition, 24 stimuli (12 familiar songs and 12 unfamiliar songs) were presented via in-ear headphones each sound type. The participants were instructed to decide whether a song excerpt was known to have been acquired in his/her life or not as quickly and accurately as possible by pressing one of two buttons: the first button, pressed with the right index finger, signified that the participant was familiar with the song, and the second button, pressed with the right middle finger, signified that the participant was unfamiliar with the song. During each trial, a warning stimulus was presented for 500 ms, followed by a silence interval (500 ms), then the target auditory stimulus (3000 ms), then silence (500 ms), followed by the next trial. The auditory stimulus was stopped immediately after a response button was pressed by the participant in order to prevent additional processing. Reaction time (RT) was measured as the interval between target stimulus onset and participant response. The designation of a song as familiar signifies that the participant had learned it well during his/her life.

The Control (sound-type decision) task was designed to recruit multiple processes, such as listening, monitoring variations in phoneme and pitch, working memory, decisional processing, and motor processing. The participants were asked to decide whether an excerpt that they heard belonged to the complete-song type (song) or to the other types (lyrics-in-isolation type [lyrics] and melody-in-isolation type [melody]) as quickly and accurately as possible by pressing one of two buttons: the first button, pressed by the right index finger, signified the song type, and the second button, pressed with the right middle finger, signified that the excerpt was one of the other types. The three types of sound stimuli were presented in a random order. Identical stimuli were used in both the FamD and the control tasks so as to balance the acoustical and perceptional processes.

The three FamD conditions (Song, Lyrics, and Melody) and the Control condition were presented to participants in a counterbalanced order. Then, each of four conditions was performed twice. RT and button selection data were recorded using a response box placed under the participant's right hand, which was linked to a computer running the E-Prime software package. In a post-scan test, participants were asked to rate the familiarity of the 78 songs presented as song, lyrics, and melody stimuli types, including the stimuli used during the PET session, using a 5-point scale.

### Data acquisition

Regional cerebral blood flow (rCBF) was measured via PET scanning using ^15^O-labeled water. A SET 2400W scanner (Shimadzu Inc., Kyoto, Japan), operated in three-dimensional mode, acquiring a 128×128×50 in matrix with a 2×2×3.125-mm voxel size. Each participant was scanned eight times to measure the distribution of ^15^O-labeled water with a 10-min inter-scan interval to allow for decay. Each scan was started upon the appearance of radioactivity in the brain after an intravenous bolus injection of 180 MBq of ^15^O-labeled water. Each scan lasted 60 s. The activity measured during this period was summed and used as a measure of rCBF. A transmission scan was obtained using a ^68^Ga/^68^Ge source for attenuation correction prior to participant scanning. Each experimental condition was started 15 s before data acquisition and continued until the completion of the scan. Participants were scanned while lying supine with their eyes closed in a darkened, quiet room. T1-weighted structural MRI scans were also obtained for each participant on a 1.5-T GE Signa system (SPGR: TR = 21 ms, TE = 6 ms, matrix = 256×256×125 voxels) for anatomical reference and in order to screen for any asymptomatic brain disorders.

### Behavioral data analysis

For each of the three types of auditory sound stimuli, the degree of familiarity measured in the post-scan task was calculated. Mean familiarities were analyzed by means of paired *t*-tests. For each participant and for each experimental condition, mean RTs were calculated. Accuracy was calculated based on the results of the post-scan familiarity rating task. We performed a repeated-measures ANOVA on RTs and accuracy. Behavioral data was analyzed by SPSS 17.0 (SPSS Inc., Chicago, USA). Post hoc, Bonferroni-corrected, paired *t*-tests were used to test for differences between conditions.

### Imaging analysis

PET images were analyzed using the Statistical Parametric Mapping software package (SPM8, Wellcome Department of Cognitive Neurology, London, UK), implemented in MATLAB 7.5.0 (Mathworks Inc., Massachusetts, USA). During preprocessing, PET data were realigned, spatially transformed into standard Montreal Neurological Institute stereotactic space (MNI, voxel size 2×2×2 mm), and smoothed with a 12-mm Gaussian filter. Each scan was scaled to a mean global activity of 50 ml/100 g/min. We used a threshold of 80% of the whole brain mean as the cutoff point for designation of voxels as containing gray matter, and covariates were centered around their means before inclusion in the design matrices. An analysis of covariance (ANCOVA), with global activity as a confounding covariate, was performed on a voxel-by-voxel basis. The results, expressed in SPM as *t*-statistics (SPM {t}), were then transformed onto a standard normal distribution (SPM {z}). All statistical thresholds were set at *p*<0.005, uncorrected at the voxel level, with an extent threshold requiring a cluster size of more than 20 voxels.

**Figure 2:**
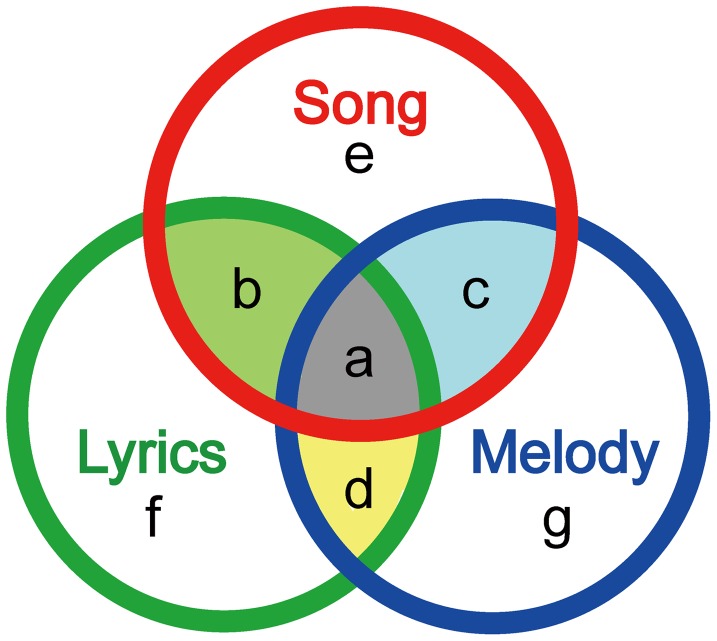
Activations observed under the contrasts (each familiarity decision task compared to the Control task) were categorized mainly into seven processes by conjunction analyses, a: common to all three, b: common to Song and Lyrics, c: common to Song and Melody, d: common to Lyrics and Melody, e: specific for Song, f: specific for Lyrics, and g: specific for Melody.

**Figure 3:**
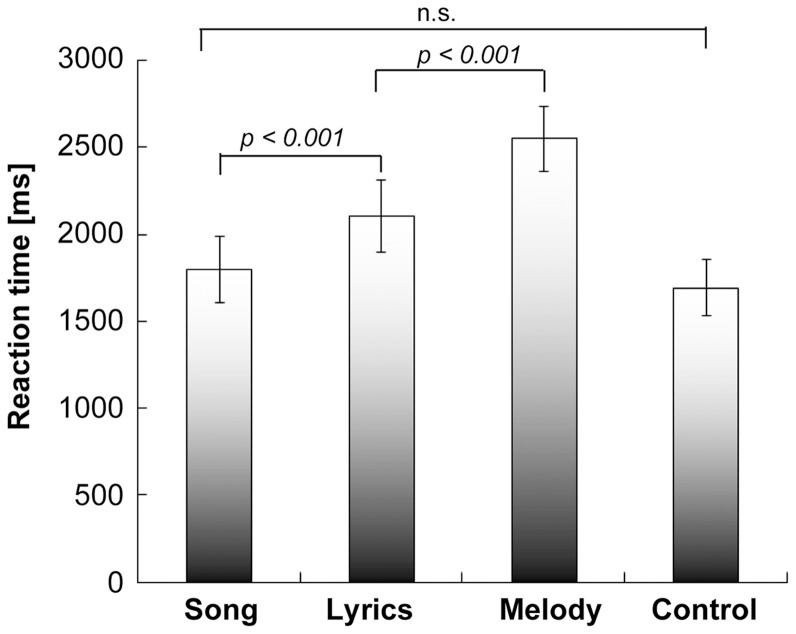
Mean reaction times for the four different conditions. Error bars indicate the standard error (*P<0.001,* Bonferroni corrected).

**Figure 4:**
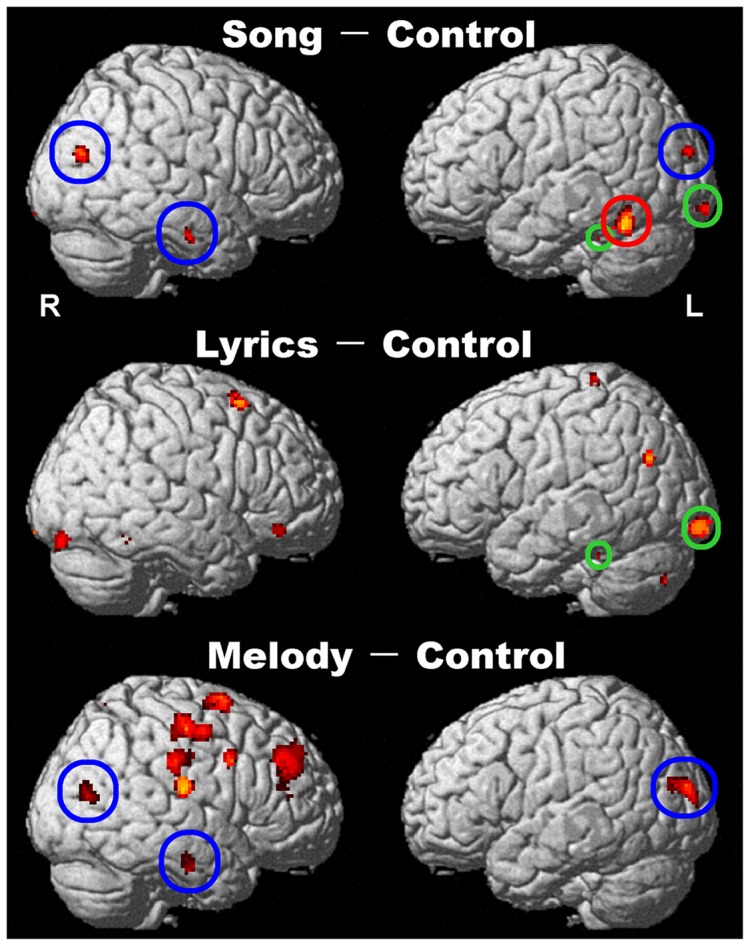
The regions activated by each familiarity decision task (Song, Lyrics, or Melody) compared to the Control task (p<0.005, uncorrected, with multiple-comparison correction, cluster size >20 voxels). As per conjunction analysis, a red circle represents specific activation for Song (Song–Lyrics ∩ Song–Melody ∩ Song–C); a green circle represents regions activated by both Song and Lyrics (Song–C ∩ Lyrics–C); a blue circle represents regions activated by both Song and Melody (Song–C ∩ Melody–C).

**Figure 5:**
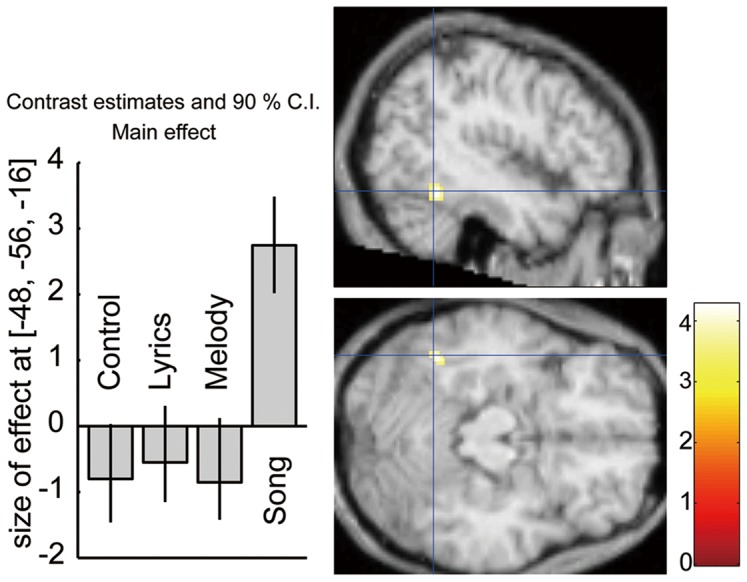
The region specifically activated by Song (Song–C ∩ Song–Lyrics ∩ Song–Melody). Number of color bar represents *z*-value. The plots represent the relative intensities in this region of interest under the different conditions. Error bars represent the standard error of the mean.

First, using *t*-tests, we created SPM contrasts subtracting the Control condition from each of the three FamD conditions: Song–C, Lyrics–C and Melody–C. Then, we performed conjunction analyses to classify the activations in each FamD condition in terms of whether they were also activated in either of the other two conditions (Figure 2). For instance, areas of activation in the contrasts (Song–C, Lyrics–C and Melody–C) may be classified into seven categories: (1) commonly activated in all three FamD conditions (Song–C ∩ Lyrics–C ∩ Melody–C, Figure 2, a), (2) commonly activated by Song and Lyrics (Song–C ∩ Lyrics–C, Figure 2, b), (3) commonly activated by Song and Melody (Song–C ∩ Melody–C, Figure 2, c), (4) commonly activated by Lyrics and Melody (Lyrics–C ∩ Melody–C, Figure 2, d), (5) specifically activated by Song (Song–Lyrics ∩ Song–Melody ∩ Song–C, Figure 2, e), (6) specifically activated by Lyrics (Lyrics–Song ∩ Lyrics–Melody ∩ Lyrics–C, Figure 2, f), and (7) specifically activated by Melody (Melody–Song ∩ Melody–Lyrics ∩ Melody–C, Figure 2, g).

## Results

### Behavioral data

Participants' degrees of familiarity with the sound stimuli in the song, lyrics and melody categories did not significantly differ (paired *t-*tests: *t* = 0.83, *p*>0.14 for song vs. lyrics; *t* = 1.51, *p*>0.14 for song vs. melody; *t* = 1.20, *p*>0.24 for lyrics vs. melody). The mean performance accuracies were 99.6% (SD = 0.59) for Song, 99.6% (SD = 0.70) for Lyrics, and 96.0% (SD = 2.18) for Melody in the FamD task and 97.2% (SD = 1.57) in the Control task. No significant differences in accuracy were observed between Song and Lyrics (*p*>0.40) or between Melody and Control (*p*>0.90), although accuracy was slightly lower for Melody and Control than for Song and Lyrics. We excluded 1.4% of all data in each condition because RTs were more than 2*SD* above the mean. A repeated-measures ANOVA showed a significant main effect of auditory stimulus type on RT (*F* (2,42) = 60.58, *p*<0.0001) (Figure 3). The mean RTs for Song were the fastest of the three types of sound stimuli in the FamD task (*p*<0.001, Bonferroni corrected) and the mean RTs for Melody were the slowest of the three types of sound stimuli. The mean RTs for Lyrics were significantly slower than those for Song but significantly faster than those for Melody. The mean RTs for Control were not different from those for Song (*p* = 0.58). We observed no significant task order effect (first vs. second) in any of the tasks (*F* (1,20) = 0.011, *p* = 0.92).

### PET activation data

In contrast to the Control, Song and Lyrics showed mainly bilateral activation patterns, whereas Melody showed right-dominant activation patterns (Figure 4 and Table 1). As expected, activations in primary and secondary auditory areas (Brodmann's areas [BA] 41, 22, and 21) were not detected due to being masked by activation in Control.

**Table 1:** Brain regions activated by the three familiarity decision conditions more strongly than by the control condition.

Contrast and anatomical location	x	y	z	Z score
***Song-Control***				
L Posterior inferior temporal cortex (BA 37)	−46	−56	−18	4.26
L Fusiform gyrus (BA 37)	−34	−40	−24	3.03
L Medial frontal gyrus/CG (BA 32)	−2	40	−12	3.65
L Inferior frontal gyrus (BA 47)	−38	32	−36	3.30
L Precuneus (BA 30)	−16	−56	20	3.28
L Cuneus (BA 7/19)	−12	−84	24	3.10
L Temporo-occipital cortex (BA 19)	−28	−90	22	3.01
R Temporo-occipital cortex (BA 39)	36	−76	20	3.10
R Middle temporal sulcus (BA 20)	50	−14	−28	3.01
L Inferior occipital gyrus (BA 18)	−22	−102	−12	3.00
***Lyrics-Control***				
L Medial frontal gyrus/CG (BA 32/10)	−6	38	−12	4.01
L Globus pallidus	−12	−6	−8	4.20
R Middle frontal gyrus (BA 8)	34	14	62	4.10
R Anterior insula (BA 13)	32	16	14	3.35
R Inferior frontal gyrus (BA 47)	50	38	−12	3.06
L Anterior part of hippocampus	−28	−12	−16	3.07
L Angular gyrus (BA 39)	−40	−68	28	3.20
L Fusiform gyrus (BA 37)	−36	−40	−24	3.63
L Inferior occipital gyrus (BA 18)	−24	−96	−12	3.84
R Inferior occipital gyrus (BA 18)	32	−86	−18	3.21
L Cerebellum crus I	−26	−76	−40	3.28
***Melody-Control***				
R Supplementary motor area (BA 6)	14	2	66	5.30
L Supplementary motor area (BA 6)	−6	−26	60	3.07
R Premotor cortex (BA 6)	62	10	36	3.29
R Middle frontal gyrus (BA 9)	26	38	34	4.41
L Middle frontal sulcus (BA 6)	−26	−8	46	3.80
R Inferior precentral gyrus (BA 4)	68	−22	34	3.83
R Postcentral gyrus (BA 3)	40	−16	36	3.71
R Middle temporal sulcus (BA 20)	44	−16	−18	4.39
L Temporo-occipital cortex (BA 19)	−32	−90	18	4.06
R Temporo-occipital cortex (BA 39)	36	−72	18	3.14
L Precuneus (BA 7)	−8	−66	54	3.16
R Precuneus (BA 7)	12	−58	62	3.31

*Notes*: Coordinates (x, y, z) are in Montreal Neurological Institute stereotactic space. BA, Brodmann's area.

Some regions activated by Song were also activated by Lyrics or Melody (Figure 4, Table 1). According to the results of conjunction analysis, areas of activation common to the Song–C and Lyrics–C contrasts were located in the medial part of the superior frontal gyrus and the cingulate gyrus (BA 32), centered at MNI coordinates [−4, 38, −12], in the anterior part of the fusiform gyrus (BA 37) [−34, −40, −24], and in the inferior occipital cortex [−22, −100, −12], all in the left hemisphere. Areas of activation common to the Song–C and Melody–C contrasts were detected in the right temporo-occipital cortex (BA 39) [36, −74, 20], in the left temporo-occipital cortex (BA 19) [−30, −92, 22], and in the right middle temporal sulcus (BA 20) [50, −14, −28]. No activations common to the Lyrics–C and Melody–C contrasts were detected by conjunction analysis. Accordingly, no areas of activation were common across all three FamD conditions compared to the Control condition.

We observed a region activated specifically in Song, but not to Lyrics or Melody, located in the posterior portion of the left inferior temporal cortex (PITC) (Figure 5). Lyrics specifically activated areas in the ventral portion of the right inferior frontal gyrus, the right anterior insula, the left angular gyrus, and the cerebellum, whereas Melody specifically produced right-dominant frontal-parietal activations (Table 2).

**Table 2:** Brain regions specifically activated by one component of songs (Lyrics or Melody).

Contrast and anatomical location	x	y	z	Z score
***Lyrics-Song*** ** ∩ ** ***Lyrics-Melody*** ** ∩ ** ***Lyrics-Control***				
R Ventral Inferior frontal gyrus (BA 47)	50	38	−12	3.26
R Anterior insula (BA 13)	32	18	16	3.35
L Angular gyrus (BA 39)	−40	−68	32	3.12
L Cerebellum_crus I	−26	−76	−40	3.14
***Melody-Song*** ** ∩ ** ***Melody-Lyrics*** ** ∩ ** ***Melody-Control***				
R Middle frontal gyrus (BA 9)	28	38	28	3.95
R Premotor cortex (BA 6)	56	8	34	3.09
R Postcentral gyrus (BA 3 1 2)	52	−16	54	3.19
R Parietal operculum (BA 40)	64	−18	18	3.43
R Middle temporal gyrus (BA 21)	44	−18	−14	3.31

## Discussion

The main purpose of this PET study was to elucidate the neural networks responsible for the semantic memory of familiar songs. We directly compared neural activation in three familiarity decision conditions: Song, Lyrics, and Melody relative to a control condition (Control, C) so as to reveal the neural mechanisms of verbal and melodic processing involved in lexical lookup of songs.

### Behavioral results

The behavioral results showed that mean RTs in Song were significantly faster than those in Lyrics and Melody, though the three types of sound stimuli were equivalently familiar to participants and had identical temporal structures (rhythm and tempo). This result is consistent with our previous behavioral study [34], which indicates that a familiar melody can facilitate the recall of lyrics [32] and that a strong connection between lyrics and melody facilitates song recognition [34], [36]. In addition, mean RTs in Melody were significantly slower than those in Song and Lyrics. This result is consistent with the findings of a number of previous psychological studies that indicated a large advantage for words over melodies during recognition tests involving songs and melody, priming experiments, familiarity decision tasks, and gating paradigms [34], [36]–[39].

### Subtraction analyses: each of the three familiarity decision conditions compared to the Control condition (FamD–C)

As expected, no contrasts between FamD and the Control showed activations in auditory regions, including Heschl's gyrus and the superior temporal gyrus. Previous studies suggested that the roles of the superior temporal gyrus in language are related primarily to speech perception and phonological processing rather than to lexical processing [25], [40]–[42]. Therefore, the contrasts (FamD–C) may allow us to investigate which areas are preferentially involved in lexical lookup of songs beyond auditory perceptual processing [18], [22], [43].

Direct comparisons among the contrasts (Song–C, Lyrics–C, and Melody–C) showed no areas of activation common to the three types of sound stimuli because the areas of activation visualized under the contrast (Lyrics–C) showed no overlap with those visualized under the contrast (Melody–C) (Figure 4, Table 1). On the other hand, some activated regions found by the contrast (Song–C) were common to those found by the contrast (Lyrics–C) and by the contrast (Melody–C). Those common regions may represent verbal lexical processing and melodic lexical processing in song recognition, respectively. These results suggest essentially separate neural networks between verbal and melodic access to the song lexicon. This finding is consistent with previous neuroimaging studies, which demonstrated that the neural substrates of classical melodies and proverbs in semantic memory tasks are distinct [30], [31]. On the other hand, Schön et al. [14] have shown similar networks involved in lyrics and melody processes in the stage of song perception. In the present study we examined neural activations in the stage of lexical lookup of songs beyond the perceptual processing which was masked by the Control. Therefore, it is suggested that the interrelationships between lyrics and melody differs depending on the stage of song processing.

### Verbal lexical processing in song recognition (common areas of activation between Song–C and Lyrics–C)

Common areas of activation detected by conjunction analysis (Song–C ∩ Lyrics–C) were located in the left fusiform gyrus, the left inferior occipital gyrus, and the medial part of the superior frontal gyrus and cingulate gyrus. The left fusiform gyrus has been implicated not only in visual word processing but also in auditory word processing [44]–[48]. A review of activation of the fusiform gyrus suggested that words presented in the auditory modality yield activation anterior (average y = −43) to the area referred to as the visual word form area (VWFA) (average y = −60) [49], [50]. Activation in the anterior part of the left fusiform gyrus has been observed more strongly during listening to words than to pseudowords [44], and this area is also preferentially activated during listening to the voices of familiar people relative to those of unfamiliar people in speaker recognition tasks [45]. Lesion or hypoperfusion in the left fusiform cortex extending into the posterior middle and inferior temporal cortices (BA 37) has been previously associated with semantic errors in naming (anomia) without a deficit in spoken word comprehension [51]–[53]. Therefore, the results of the present study suggest that the left fusiform gyrus may be important for the lexical lookup of lyrics. The left medial part of the superior frontal gyrus and the anterior cingulate gyrus have been found to be involved in decision processing using semantic memory [27], [29], [54], [55].

### Melodic lexical processing in song recognition (common activations between Song–C and Melody–C)

Common areas of activation detected by the conjunction analysis (Song–C ∩ Melody–C) were located in the bilateral temporo-occipital cortices and the right middle temporal sulcus. Several previous studies have reported the recruitment of these temporo-occipital cortices during non-visual auditory tasks. For instance, activation in the bilateral temporo-occipital cortices was observed during listening to the soprano part of a harmony relative to that observed during listening to the harmony as a whole [56]. Activation in the left temporo-occipital cortex (BA 19) was observed during familiarity decision processes using classical melodies [19]. Activation in the right temporo-occipital cortex has been found during decisions about whether pitches are ascending or descending [57], during imagery of sequence of five notes in random order just heard before [58], and during listening or covert singing [11]. We also observed involvement of the precuneus in both the Song–C and Melody–C contrasts, although the location of the peak of activation was slightly different between Song and Melody (Table 1). The precuneus is well known for its contribution to imagery-related processes (for a review, see [59]). Our results indicate that the bilateral temporo-occipital gyri, together with the precuneus, may be implicated in the comparison of melodic contours between experimental auditory stimuli and melodies stored in memory. The right middle temporal gyrus and sulcus have been implicated as being selectively involved in the processing of semantic musical memories as opposed to episodic musical memories [27], and these areas are also more involved in the process of making familiarity decisions about melodies than in the detection of melodic alteration [28]. Therefore, the right middle temporal sulcus may be responsible for the melodic processing of lexical lookup in song recognition.

### Specific activation when all properties of song are intact (Song–Lyrics ∩ Song–Melody ∩ Song–C)

One of our main findings is that the left PITC (BA 37) selectively responded to Song, but not to Lyrics, Melody, or Control (Figure 5). The location of the activation peak in this area was [−48, −56, −16], a location slightly lateral and posterior to the fusiform gyrus [−34, −40, −24], which is commonly activated in Song–C and Lyrics–C. Although the left PITC (BA 37) has often been considered to be part of the visual association cortex [25], [54], [60], [61], several functional imaging studies have observed that this region is activated by auditory verbal stimuli in a variety of lexical tasks [31], [46], [48], [62]–[65]. It is suggested that the left PITC [−42, −54, −16] acts as an interface between visual form information and its associated sound and meaning [61]. In the dual-stream model of speech processing [21], [66], regions including the PITC has been nominated as part of the ventral stream; it appears to be involved in mapping auditory phonological representations onto lexical conceptual representations. Moreover, recent studies have reported that the left posterior inferior temporal region (BA 37) plays a central role in the multisensory representation of object-related information across the visual, auditory, and/or tactile modalities [67]–[69]. Therefore, we suggest that in the present study, the left PITC (BA 37) plays an important role in lexical lookup of songs as an interface region between lyrics and melody. The specific activation by Song in this area could help to explain why the mean RTs in Song were faster than those in Lyrics and Melody.

### One component (Lyrics or Melody) evoked more activation than Song or Control

We found the neural distinction between Lyrics–C and Melody–C (Figure 4). The conjunction analysis (Lyrics–Song ∩ Lyrics–Melody ∩ Lyrics–C) identified activations in the right ventral inferior frontal gyrus (BA 47) and the right anterior insula (Table 2) as being specific to Lyrics. These right-hemispheric structures have been reported to be responsible for the retrieval and imagery of melody [58], [70], [71], for singing [17], [72], and for the discrimination of pitch [73], [74]. Therefore, our results indicate that the right inferior frontal gyrus and the right anterior insula may be responsible for the retrieval of an appropriate melodic contour to compensate for flattened pitch. In addition, we observed that Lyrics elicited specific activation in the left angular gyrus. The left angular gyrus has been reported to be involved in speech comprehension tasks [75]–[78]. Thus, it is suggested that the stimuli in Lyrics may make greater demands on verbal comprehension in song recognition tasks relative to the stimuli in Song, in which melodic components were intact.

The conjunction analysis (Melody–Song ∩ Melody–Lyrics ∩ Melody–C) showed that activations specific to Melody were widely distributed in the right fronto-parietal regions, including the premotor and somatosensory areas (Table 2). These regions have been reported to be involved in the retrieval, imagery, working memory storage, and rehearsal of melodies and in the imagery of singing [58], [70], [79]. Taken together, our results suggest that Melody demands motor strategies such as auditory-to-articulatory mapping so as to maintain the information gleaned by excerpts, retrieve the next part of the song from memory, and identify melodic lines for song recognition [14], [80].

One limitation of our study is that we were not able to fully control the effect of automatically accessing the song lexicon. We merely used identical stimuli in the FamD and the Control in order to balance the perceptual processing across conditions. Thus, we cannot rule out the possibility that the Control automatically evokes the execution of lexical lookup processes. This might be the reason why we did not detect activation in the superior temporal sulcus, which has been implicated in automatic access to the musical lexicon [22]. Further studies are needed in order to elucidate the neural mechanisms of musical semantic memory, focusing on the automatic lexical lookup of songs.

## Conclusions

The present study investigated the neural substrates responsible for the verbal and melodic processes involved in the semantic memory of familiar songs. Our results demonstrate essentially separate neural networks controlling verbal processing and melodic processing during lexical lookup of songs. The verbal representation recruited the left fusiform gyrus and the left inferior occipital gyrus, whereas the melodic representation engaged the right middle temporal sulcus and the bilateral temporo-occipital cortices. Moreover, we found that the left PITC (BA 37) was specifically activated by feature-complete songs, but not for lyrics or melody. The left PITC appears to play a crucial role in song lexical lookup of songs as an interface area between lyrics and melody to facilitate the recognition of familiar songs.
